# Event-to-event intensification of the hydrologic cycle from 1.5 °C to a 2 °C warmer world

**DOI:** 10.1038/s41598-019-39936-2

**Published:** 2019-03-05

**Authors:** Gavin D. Madakumbura, Hyungjun Kim, Nobuyuki Utsumi, Hideo Shiogama, Erich M. Fischer, Øyvind Seland, John F. Scinocca, Daniel M. Mitchell, Yukiko Hirabayashi, Taikan Oki

**Affiliations:** 1 Department of Civil Engineering, The University of Tokyo, Tokyo, Japan; 2 Present Address: Department of Atmospheric and Oceanic Sciences, University of California, Los Angeles, Los Angeles, CA USA; 3 Institute of Industrial Science, The University of Tokyo, Tokyo, Japan; 4 Jet Propulsion Laboratory, California Institute of Technology, Pasadena, CA USA; 5 Center for Global Environmental Research, National Institute for Environmental Studies, Tsukuba, Japan; 6 Institute for Atmospheric and Climate Science, ETH Zurich, Universitätstrasse 16, 8092 Zurich, Switzerland; 7 Norwegian Meteorological Institute, Oslo, Norway; 8 Canadian Centre for Climate Modelling and Analysis, Environment and Climate Change Canada, University of Victoria, Victoria, V8W 2Y2 Canada; 9 School of Geographical Sciences, University of Bristol, Bristol, UK; 10 Department of Civil Engineering, Shibaura Institute of Technology, 3-7-5 Toyosu, Koto-ku, Tokyo, Japan

## Abstract

The Paris agreement was adopted to hold the global average temperature increase to well below 2 °C and pursue efforts to limit it to 1.5 °C. Here, we investigate the event-to-event hydroclimatic intensity, where an event is a pair of adjacent wet and dry spells, under future warming scenarios. According to a set of targeted multi-model large ensemble experiments, event-wise intensification will significantly increase globally for an additional 0.5 °C warming beyond 1.5 °C. In high latitudinal regions of the North American continent and Eurasia, this intensification is likely to involve overwhelming increases in wet spell intensity. Western and Eastern North America will likely experience more intense wet spells with negligible changes of dry spells. For the Mediterranean region, enhancement of dry spells seems to be dominating compared to the decrease in wet spell strength, and this will lead to an overall event-wise intensification. Furthermore, the extreme intensification could be 10 times stronger than the mean intensification. The high damage potential of such drastic changes between flood and drought conditions poses a major challenge to adaptation, and the findings suggest that risks could be substantially reduced by achieving a 1.5 °C target.

## Introduction

The Paris agreement of 2015 was adopted at the 21st Conference of the Parties of the United Nations Framework Convention on Climate Change following concurrence to hold the global average temperature increase to well below 2 °C and pursue efforts to limit it to 1.5 °C above pre-industrial levels by the year 2100^1–3^. Since then, climate scientists have been engaging in efforts to investigate the impacts of an additional half degree warming from 1.5 °C to 2 °C. After a tremendous effort, a special report was produced by the Intergovernmental Panel on Climate Change (IPCC) on the impacts and greenhouse gas emission pathways related to 1.5 °C global warming target^4^. Global warming is highly likely to surpass 1.5 °C target under emission scenarios based on current policies and strengthened climate actions than current pledges made under the Paris Agreement will be required to limit the global warming to 1.5 °C^5,6^. Studies have already discussed the impacts of limiting the warming to 1.5 °C in many areas of earth system sciences^7–10^. However, changes in many aspects of natural phenomena on Earth are still uncertain between a 1.5 °C and 2 °C target, and such changes need to be quantified.

Human-induced global warming has contributed to an increase in the magnitude and frequency of climate extremes^11^. Global warming has the potential to change the frequency and the intensity of precipitation events by intensifying the hydrologic cycle^12–16^. Precipitation change can manifest itself as the rain events becoming more frequent and more intense, more frequent and less intense, or less frequent and more intense^17^. This can lead to a hydroclimatic intensification with increased consecutive dry days or increased precipitation intensity, or both^16,17^. From a thermodynamic perspective, this hydroclimatic intensification is mainly linked to the increase in the atmospheric water holding capacity according to the Clausius–Clapyeron (C-C) relation, the increase in evapotranspiration with rising temperatures and the imbalance in the rate of increase of these variables^16^. By using global and regional climate model experiments, studies have shown that intensification of the water cycle is a consistent and ubiquitous signature of 21st century greenhouse-induced global warming for medium to high warming scenarios^16,17^. However, such hydroclimatic intensification assessments for lower warming targets such as 1.5 °C and 2 °C have not been conducted yet.

In regard to the daily precipitation, there are days with precipitation (wet spells) and days where no significant precipitation occur (dry spells). The number of these wet and dry spells and their severities are naturally interconnected and potentially related to extreme hydroclimatic events such as droughts and floods. Droughts are naturally associated with sustained periods of little to no precipitation, i.e., extended dry spells. Flood events can arise though short intense storms, and also from continuous periods of heavy or moderate precipitation, which correspond to intensified and/or extended wet spells. Intensification of adjacent dry and wet spells together has the potential to transform conditions into prolonged droughts followed by extreme flooding and vice versa, such as the switch from extreme drought to severe flooding that occurred in California during the recent past^18^. Such events are even suggested to be increased in California in a higher global warming scenario in an inter-annual context^19^. However, the changes in the frequency and intensity of wet and dry spells and their interconnectivity at a sub-seasonal to seasonal scale, which can form adverse conditions, are not well understood.

This study was conducted to investigate the global water cycle intensification, with an emphasis on changes in the intensity and frequency of wet and dry spells, which can be expected in 1.5 °C and 2 °C warmer worlds. Analyses were performed at the intra-annual scale, and extreme conditions were assessed as well. For the analyses, we utilized four atmospheric general circulation model (AGCM) experiments from the project titled “half a degree additional warming, prognosis and projected impacts” (HAPPI)^2,3^. With the models MIROC5, NorESM, CanAM4, and CAM4, three sets of scenarios were employed, namely, a historical scenario for the period 2006–2015 (ALL) and 1.5 °C and 2 °C equilibrium warming scenarios for a 10-year period in the beginning of 22nd century (hypothetically for the 2106–2115 period). Daily precipitation output from 100 ensemble members per scenario per model was used. Inspired by an earlier work^16^, here, we propose the “event-to-event hydrological intensification index” (E2E), which combines normalized “aggregated precipitation intensity” (API) and “dry spell length” (DSL), to capture the interconnectivity of adjacent dry and wet spells and the intensification of their phase shifts (see Methods for more details). Governing processes that change the wet and dry spell intensity and frequency are likely to be interconnected^12,14,16^. This will result in changes of DSL and precipitation intensity in an interrelated manner. In an intensified hydrologic cycle, either both variables will increase when the mean precipitation does not change significantly, or the increase in one variable will overwhelm the change in the other when the mean precipitation changes^16^. E2E provides an integrated assessment of these variables and such assessments of hydroclimatic intensification have been demonstrated to give ubiquitous, and enhanced signals of the hydrologic cycle’s response to global warming than individual metrics such as the DSL and precipitation intensity^16,17^.

**Figure 1:**
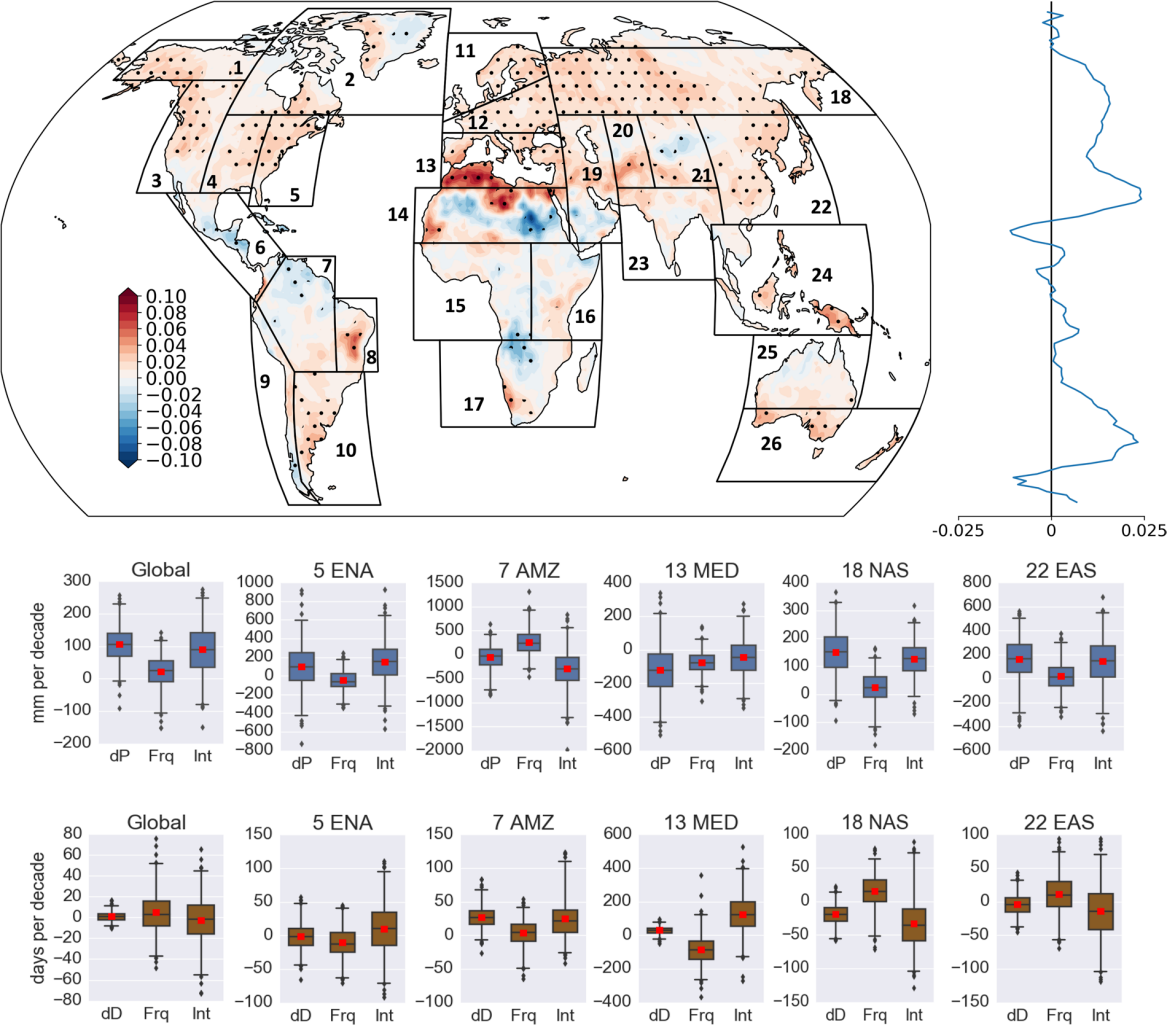
Global spatial map and zonal mean of the 2 °C minus 1.5 °C E2E multi-model ensemble mean of the HAPPI data^3^ (95% significant level is stippled). Black boxes represent the IPCC AR5 reference regions (http://www.ipcc-data.org/guidelines/pages/ar5_regions.html). Box-whisker plots show the area averaged (land only) difference between 2 °C and 1.5 °C climates (2 °C minus 1.5 °C) for the total precipitation (dP), frequency term (Frq), and intensity term (Int) during wet spells in mm per decade (in the middle panel) globally and over the East North America (ENA), Amazon (AMZ), South Europe/Mediterranean (MED), and East Asia (EAS) regions. The bottom panel is the same as the middle panel but for dry spells where dD is the total number of dry days and the unit is dry days per decade. Box-whisker plots indicate the 10th, 25th, 50th, 75th, and 90th percentiles, and the other points are outliers.

Figure 1 shows the multi-model mean change of the E2E between 2 °C and 1.5 °C climates. Probability density functions indicate that there will be a clear increase in the E2E with warming (Fig. S5). The zonal mean indicates that the tropics will face a weakening of event-to-event variability, while mid latitudes will experience a peak, which disappears in the high latitudes. For the additional 0.5 °C warming, a significant decrease in the E2E can be seen over the eastern part of Greenland, Central America, Amazon, Sahara, and East, and South Africa, as well as the Tibetan Plateau. North America, North East Brazil, Southeastern South America, the Mediterranean region, Europe, North, East, and South East Asia, and Southern Australia show a significant increase in the E2E. To understand the changes in total precipitation and dry days during wet and dry spells, the changes were decomposed into the changes in the intensity and the frequency of the spells (see Methods). The box-whisker plots in Fig. 1 show the multi-model ensemble results for the regional mean calculated for each ensemble. Globally, the ensemble mean precipitation showed a total increase of around 110 mm per decade (equivalent to 11 mm per year), which is about a 1.5% increase from 1.5 °C ensemble mean. This increase in precipitation amount is about 1.4% of recent estimations of the annual mean terrestrial precipitation^20^. Change in frequency of wet spells contributed to 20 mm of that change and the increase in the API contributed to the rest. The multi-model ensemble range of the precipitation change was mostly positive. Total dry days during dry spells showed no apparent change with a small multi-model ensemble range, and this finding was suggestive of a good model agreement. Frequency of dry spells was found to increase slightly while contributing to an increase of around 5 days per decade, but the decrease in the DSL compensates for that. Here, we use regional domains from the IPCC special report on extremes (IPCC SREX regions)^21^ to investigate regional changes in the East North America (ENA), Amazon (AMZ), South Europe/Mediterranean (MED), North Asia (NAS), and East Asia (EAS) regions. Results for the other SREX regions are included in the Supplementary Materials. With the additional 0.5 °C warming, the AMZ domain averaged mean precipitation showed a decrease and the number of dry days showed an increase. The number of events increased with shorter wet spells and extended dry spells, and both the intensity (DSL) and frequency terms contributed to the increase in total dry days. The decrease in the API was dominant in the AMZ compared to the increase in the DSL, which in turn led the decrease in the E2E. Central America behaved fairly similar to the AMZ (Fig. S3). An area with a significant decrease in the E2E was detected in the Southeastern part of Western Africa and the area north of South Africa, where there were increasing numbers of events with a decrease in the mean wet spell length and mean precipitation and therefore a decreasing API. A significant decrease in the DSL further contribute to the decrease of the E2E. The HAPPI multi-model ensembles have shown that the West African region will experience a decrease in the rainy season length with the additional 0.5 °C warming, which potentially is due to an anomalous migration of the Intertropical Convergence Zone towards the northern equatorial Atlantic region^22^, which is consistent with the decrease in wet spell lengths observed here. Among the regions with a significant increase in the E2E, the ENA experienced an increase in the API. The mean precipitation increased by about 100 mm per decade with no apparent change in the mean total dry days. The decrease in wet spells contributed to a slight decrease in precipitation with a dominating increase in the mean API of about 150 mm. The decrease in dry spell occurrence and increase in mean DSL of about 10 days per decade have offset each other. The significant increase in the E2E in the MED region with the additional 0.5 °C warming was caused by an increase in the DSL along with a decrease in the API. This behavior was common for other Mediterranean climate regions in South America, South Africa, and Australia. In the MED region, total mean precipitation decreased by about 100 mm per decade, while total mean dry days increased by about 20 days per decade. The inter-model ensemble spread for the change in total dry days was smaller, which suggests a robust signal. Occurrence of events decreased with shorter wet spells and longer dry spells, and therefore, a decrease (increase) in the API (DSL) was observed. This could be due to the increased moisture divergence owing to the establishment of quasi-stationary subtropical high-pressure systems with the warming, which has the potential to increase dry days and to decrease precipitation frequency in Mediterranean climate regions^23^. The EAS region showed a significant increase in the E2E due to the increase in the API even with the DSL decreasing. The mean total precipitation increased by about 180 mm per decade, mainly due to the increase in the API. This increase in the API in the Asian–Australian monsoon regions can be attributed to the increase in summer monsoon precipitation shown in the HAPPI AGCMs^24,25^. Total mean dry days decreased slightly where the decrease from the DSL change governed compared to the increase from the frequency term. The NAS region behaved similar to the EAS region under the warming climate.

**Figure 2:**
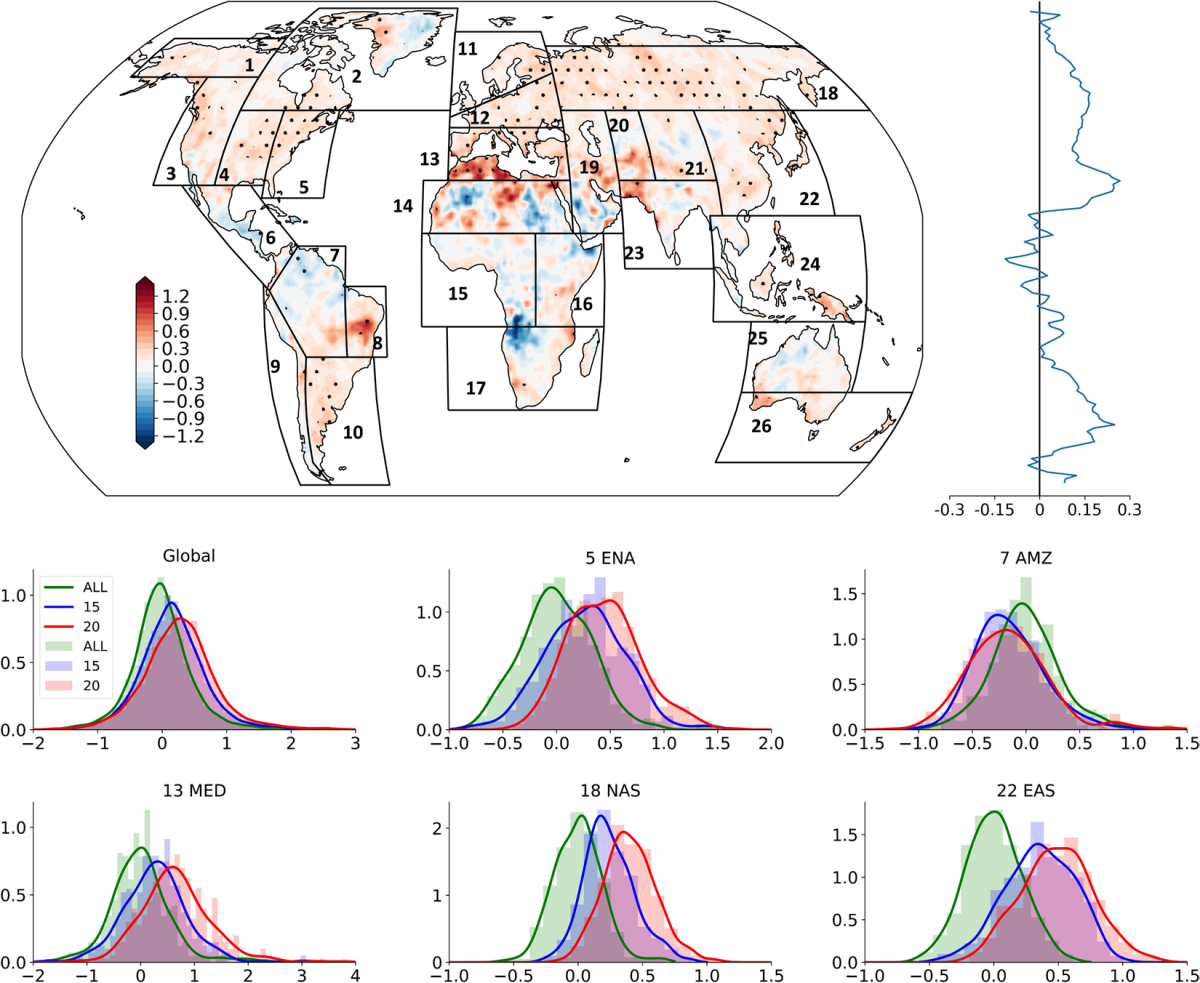
Global spatial map and zonal mean of the 2 °C minus 1.5 °C E2E multi-model ensemble 99th percentile (P99) of the HAPPI data^3^ (95% significant level is stippled). Black boxes represent the IPCC AR5 reference regions (http://www.ipcc-data.org/guidelines/pages/ar5_regions.html). Histograms and kernel density estimations (KDE; thick line) are of the area averaged (land only) E2E P99 distribution in each region for the historical (ALL) period and 1.5 °C (15) and 2 °C (20) future scenarios globally and over the East North America (ENA), Amazon (AMZ), South Europe/Mediterranean (MED), and East Asia (EAS) regions. The x-axis shows the E2E P99 values, and the y-axis shows the probability. ALL, 1.5 °C, and 2 °C scenarios are shown by green, blue, and red colors, respectively. The bin width of the P99 histograms was set to 0.1.

Extreme conditions of the cases for the E2E change with the warming are shown in Fig. 2 as the 99th percentile value (P99). Spatial patterns and the zonal mean distribution of the E2E P99 were very similar to those of E2E mean. Spatially, the change due to 0.5 °C warming is about 10 times in P99, compared to the mean. However, the area fraction with a significant difference shows a reduction, globally. Peak values of the probability distribution of the global mean of P99 anomalies in 1.5 °C and 2 °C climates increased around 10-fold compared to the mean E2E (Fig. S5). Mean of the P99 anomaly distribution increased by about 63%, globally, from 1.5 °C to 2 °C (Tables S1 and S2). A higher skewness is an indicator of an increase in tail end values of the distribution which could occur through increased extreme DSL or API or both. For instance, regions where a change in DSL, have a higher contribution to the change in E2E, such as MED, are suggested to have higher extreme events with larger DSL, when they have an increased positive skewness in E2E P99 anomaly distribution. This is consistent with changes shown in P99 of DSL and API (Fig. S6). A statistically distinguishable (p value < 0.01) clear positive shift in P99 anomaly distributions can be seen between 1.5 °C and 2 °C globally and regionally for many regions such as ENA, MED, NAS, and EAS. The AMZ region with the decreasing E2E experienced a decrease in the peak for 2 °C compared to that for 1.5 °C and an elongated negative tail where 2 °C results had a higher frequency for E2E range from −0.6 to −1.0.

This study demonstrates that intensification of the hydrologic cycle will occur with the projected warming, and the emphasis was placed on the sub-seasonal to seasonal variability of combined wet and dry spell characteristics for the additional half degree warming from 1.5 °C to 2 °C. Although some regional studies argued coupled climate ocean–atmospheric internal variability can be important for simulating realistic extreme conditions such as drought^26,27^, the utilization of multiple models and large ensemble experiments, which has merits such as reduced individual model inherent uncertainties and incorporation of large natural variability, represented the global patterns in accord with previous studies^9,17^. Based on the multi-model large ensemble AGCM experiments, we showed that warming from 1.5 °C to 2 °C will cause an escalation in the intensification of event-to-event variability in terms of magnitude. The results presented here clearly suggest extreme dry and wet events will increasingly co-occur in an event such as the switch from extreme drought to severe flooding in California during the recent past^18^, and most recently, the 2018 flood in Japan, which was followed by one of the most intense heatwaves the country has ever faced. At least, in terms of disaster mitigation and water security, there would be significant benefits to limiting global warming to 1.5 °C to dampen the intensified event-to-event variability to which our society will likely be exposed more frequently under the business-as-usual warming.

## Methods

### HAPPI simulations

We used MIROC5, NorESM, CanAM4, and CAM4 models and three sets of scenarios, namely, a historical scenario (for the 2006–2015 period) and 1.5 °C and 2 °C equilibrium warming scenarios (for a 10-year period in the beginning of 22nd century. Hypothetically for the 2106–2115 period). ALL scenario is forced by observations. Forcing and boundary conditions of the 1.5 °C warming scenario corresponds to those of the year 2095 of the representative concentration pathway (RCP) 2.6 of Coupled Model Intercomparison project Phase 5^28^. Similar conditions are used for the 2 °C warming scenario, except for greenhouse gases, sea surface temperature and sea ice forcing, which are taken as a weighted combination of RCP2.6 and RCP4.5 scenarios. Further details are given in the HAPPI overview paper^3^.

### Derivation of E2E and the utilization of the HAPPI AGCMs

We derived the “event-to-event hydrological intensification index” (E2E) as follows. First, wet and dry days were demarcated by using the precipitation threshold 1 mm/day. We defined each consecutive wet spell and dry spell as a single event (Fig. S1). The number of these events can change temporally and can represent intra-annual conditions, which will reflect the event-to-event intensification. For each event, we calculate the dry spell length (DSL) as the consecutive number of dry days and the total daily precipitation during the wet spell, which is called the “aggregated precipitation intensity” (API) throughout this study. The E2E is the event-to-event intensification index. The DSL and API values were normalized by their 10 year historical (i.e., the ALL simulation) annual average before calculating the E2E (Eq. 1). The mean of the API can be computed by Eq. 2. Here, P is the annual total precipitation during wet days and n_w_ is the number of wet spells, which is equal (or different by 1) to the number of events (number of dry spells).

1 $${\rm{E2E}}={\rm{API}}\times {\rm{DSL}}$$

2 $${{\rm{API}}}_{{\rm{mean}}}=(\frac{{\boldsymbol{P}}}{{{\boldsymbol{n}}}_{{\boldsymbol{w}}}})$$

In Fig. S1, we demonstrate the derivation of the event-wise E2E by combining spell 1 with 2, 3 with 4, and so on. We further checked the sensitivity of the E2E by shifting the position of one spell, i.e., by combining 2 with 3, 4 with 5, and so on (will use the term E2E#2 for this from hereon). By using the global GPCP-1DD daily precipitation data set^29^ for the period 1 October 2006–1 October 2015, we derived the observed DSL, API, E2E, and E2E#2. Fig. S1 shows the E2E and E2E#2 results, which indicate that for a 10 year period, they will give similar results for the mean conditions.

Daily precipitation output from 100 ensemble members per scenario of each model was used. Initially, the event-wise DSL, API, and E2E were calculated for each ensemble (i.e., for 10 years) in their original model resolution. For the analysis of the extreme cases of hydroclimatic intensity, the 99th percentile (P99) of E2E was then obtained along with the DSL and API components of that event. This resulted in 100 values for each parameter (i.e., P99 of E2E, etc.) per model per experiment (ALL, 1.5 °C, and 2 °C). Before combining these parameters for the multi-model analysis, results were regridded into a 1-degree resolution and concatenated to calculate the multi-model data (i.e., 400 values per experiment for each grid). When deriving the probability density distributions, to remove the model inherent biases for each experiment of each model, the ensemble mean value of the ALL experiment was removed before regridding and concatenating. For instance, in the P99 E2E values of the ALL, 1.5 °C, and 2 °C experiments with the MIROC model, the ensemble mean of ALL from the same model was deducted from all values. Afterward, the anomalies were obtained. Comparison between modeled and observed variables are shown in Fig. S2.

### Intensity-frequency decomposition

The total change in the wet day precipitation (total dry days) during each decade of each ensemble was investigated in the context of the frequency and intensity of the wet (dry) events. Here, the frequency is the number of wet/dry spells and the intensity is the API (DSL) for wet (dry) spells. For wet spells, frequency–intensity decomposition is as follows. If the total precipitation (P) can be represented as a combination of the mean precipitation intensity (I, that is the mean API for wet spells) and mean frequency (n), i.e., as P = n.I, then change in the total precipitation from 1.5 °C to 2 °C warming can be decomposed as follows:

3 $${\rm{\Delta }}{\rm{P}}={\rm{P}}^{\prime} \mbox{--}{\rm{P}}=({\rm{n}}+{\rm{\Delta }}{\rm{n}}).({\rm{I}}+{\rm{\Delta }})\mbox{--}{\rm{n}}.{\rm{I}}={\rm{\Delta }}{\rm{n}}.{\rm{I}}+{\rm{n}}.{\rm{\Delta }}{\rm{I}}+{\rm{\Delta }}{\rm{n}}.{\rm{\Delta }}I$$

P′ is the precipitation under 2 °C conditions, and P, n, and I are the parameters under 1.5 °C conditions; Δ represents the change between 1.5 °C and 2 °C climates. Here, the Δn.I term represents the change due to the frequency change and n.ΔI represents the change due to the intensity change. Δn.I and n.ΔI will be called the frequency term and intensity term from now on^30^. This decomposition was conducted for precipitation larger than 1 mm/day (i.e., precipitation during wet days) in warming scenarios 1.5 °C and 2 °C. For dry spells, we can replace P with the total dry days (D) and I is equal to the mean DSL. We found that the covariance term was negligible during our analysis (Fig. S3).

### Significance tests

Two-tailed Student’s t-test was applied to calculate the statistical significance shown in spatial figures of Figs 1, 2 and Fig. S3. Assessment of the statistical significance of probability density functions were conducted using two-sided Kolmogorov-Smirnov test.

## Supplementary information

Supplementary Information
