# Supplementary material for: Event-to-event intensification of the hydrologic cycle from 1.5 °C to a 2 °C warmer world
Source: Sci Rep. 2019 Mar 5;9:3483. doi: 10.1038/s41598-019-39936-2 (PMC6400949; doi:10.1038/s41598-019-39936-2)
Supplement: Supplementary file 1 — Supplementary Information [file 41598_2019_39936_MOESM1_ESM.docx]

**Event-to-event intensification of the hydrologic cycle from 1.5°C to a 2°C warmer world**

**Gavin D. Madakumbura^1, 2^, Hyungjun Kim^3*^, Nobuyuki Utsumi^4^, Hideo Shiogama^5^, Erich M. Fischer^6^, Øyvind Seland^7^, John F. Scinocca^8^, Daniel M. Mitchell^9^, Yukiko Hirabayashi^10^, and Taikan Oki^3^**

^1^Department of Civil Engineering, The University of Tokyo, Tokyo, Japan

^2^Present address: Department of Atmospheric and Oceanic Sciences, University of California, Los Angeles, Los Angeles, CA, USA

^3^Institute of Industrial Science, The University of Tokyo, Tokyo, Japan

^4^Jet Propulsion Laboratory, California Institute of Technology, Pasadena, CA, USA

^5^Center for Global Environmental Research, National Institute for Environmental Studies, Tsukuba, Japan

^6^Institute for Atmospheric and Climate Science, ETH Zurich, Universitätstrasse 16, 8092 Zurich, Switzerland

^7^Norwegian Meteorological Institute, Oslo, Norway

^8^Canadian Centre for Climate Modelling and Analysis, Environment and Climate Change Canada, University of Victoria, Victoria, V8W 2Y2, Canada

^9^School of Geographical Sciences, University of Bristol, Bristol, UK

^10^Department of Civil Engineering, Shibaura Institute of Technology, 3-7-5 Toyosu, Koto-ku, Tokyo, Japan

^*^correspondence e-mail address: hjkim@iis.u-tokyo.ac.jp

**Figure. S1.** Schematic diagram of event wise derivation of the event to event variability index (E2E, top). Bottom panels show the spatial maps of mean E2E calculated for 2006-2015 for GPCP observed data. a) E2E of event derived by combining events 1 and 2, 3 and 4, and so on. b) E2E of event derived by combining events 2 and 3, 4 and 5, and so on.


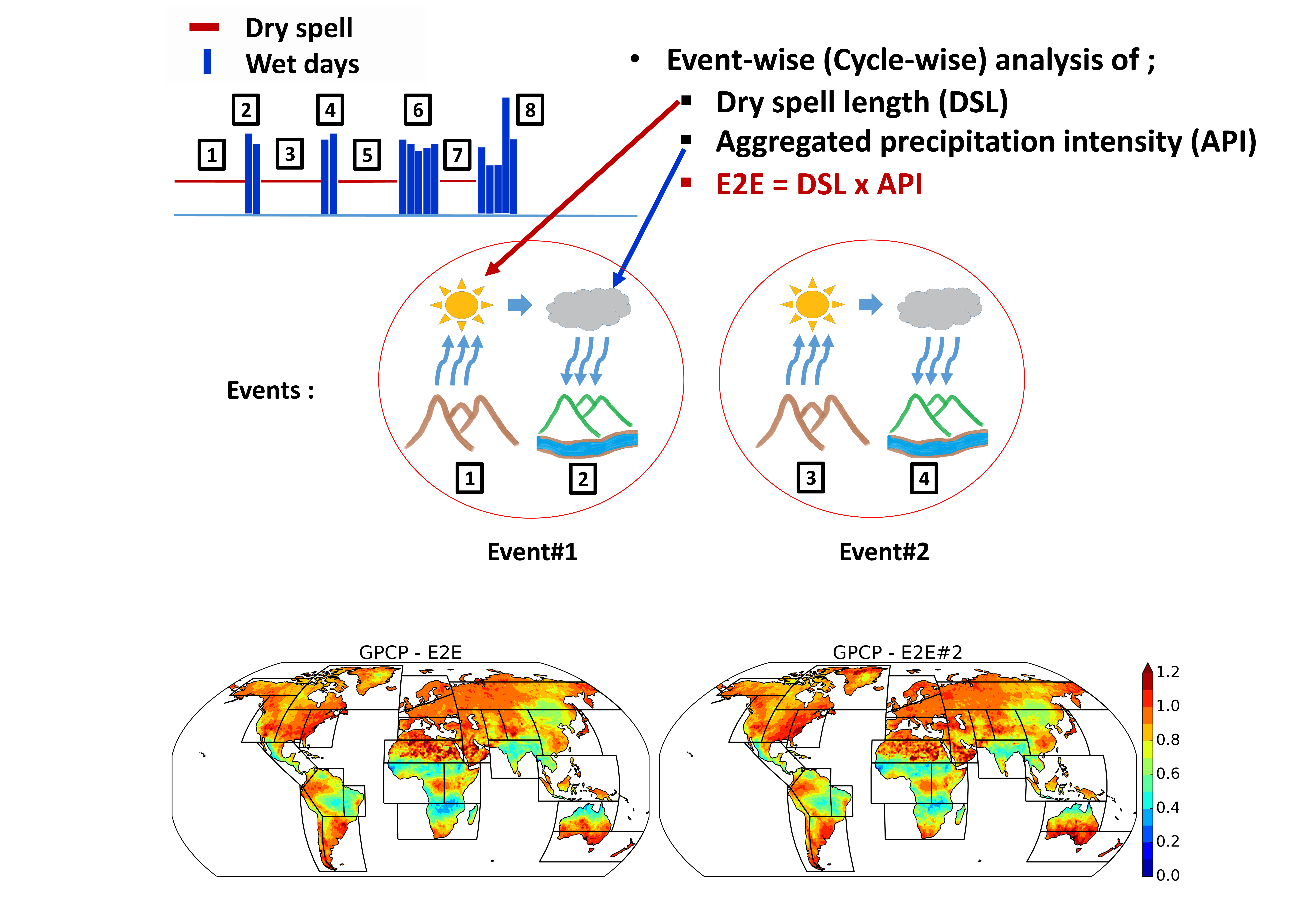


**Figure S1.** Schematic diagram of event wise derivation of the event to event variability index (E2E, top). Bottom panels show the spatial maps of mean E2E calculated for 2006-2015 for GPCP observed data^1^. a) E2E of event derived by combining events 1 and 2, 3 and 4, and so on. b) E2E of event derived by combining events 2 and 3, 4 and 5, and so on.

**Observed and modelled indices**

Observed DSL, API and E2E values calculated from GPCP-1DD daily precipitation data set^1^ were compared with the corresponding HAPPI^2^ ALL simulation multimodel ensemble mean variables (Fig. S2). DSL values in arid and semi-arid regions are well reproduced by the models. Multimodel ensemble mean DSL underestimates the intensity over Australia, Greenland and Southern part of the western North America and overestimates over north part of southern South Africa and south part of the Amazon region. Fig. S2c shows that the models capture the frequency of low dry spell lengths (e.g. 0-20 weeks) well but overestimate the frequency of longer dry spells. Fig. S2d shows the API calculated for the decade 2006-2015 using observations. It can be clearly seen that the regions with higher API values overlap with the global monsoon precipitation domains^3,4^. This is due to the fact that monsoon dominated regions have long wet spells (hence a lower value of number of wet spells, *n_w_*) associated with a substantial amount of precipitation. This is further supported by the Köppen climate classification where their tropical regions (i.e. tropical rainforest, monsoon and wet and dry savanna) regions overlap with high API values and dry regions (i.e. desert, steppe), where both *P* and *n_w_* are low, corresponds to low API values in fig S2c (ref. 5, their figure 1a). HAPPI multimodel ensemble mean overestimates the frequency of higher values of API. This is consistent with a previous study^6^ where HAPPI models used here showed a wet bias for summer monsoon regions over Asia and Australia. AGCMs can reproduce these drought-related systematic biases due to the missing coupled climate ocean-atmospheric internal variability^7,8^. Even with pronounced regional biases, multimodel ensemble mean of DSL and API show a reasonably good agreement in broad spatial patterns. Modelled E2E however shows a better agreement with observed mean (Fig S2g-i). Note that the derivation of E2E is the event-wise multiplication of normalized DSL and API. Therefore, an E2E values closer to 1 suggest a near normal DSL and API combinations. An E2E value lower (higher) than 1 corresponds to a mean of events with combinations of lower (higher) than normal DSL and/or API. For the ALL period, a lower mean E2E value can be observed in Africa, South Asia, East Asia, North Australia and, Central America, Amazon and North East Brazil. This good agreement between modelled and observed E2E suggests that in spite of the regional biases of modelled API and DSL observed earlier, daily scale intensification compared to the mean state has been captured well.

To verify the historical change in E2E (called delta E2E from hereon), an extended version from the historical simulation was compared to an observational dataset. Only the models MIROC5, NorESM and CAM4 were available, and each model provided 10 ensembles with a common timespan for 1979-2015. For periods 2001-2010 (00s) and 1979-1988 (80s), E2E was calculated for each ensemble member of the above three models. For each model, 00s minus 80s E2E was calculated for 100 possible combinations of 10 ensembles, per model. Observed E2E change was obtained from GSWP3 daily precipitation data^9^. Fig S2(j) shows that the aggregated observed delta E2E values are within the ensemble range of modeled delta E2E. However, it should be noted that the E2E difference of historical periods is strongly affected by the internal climate variability, in models and observations, irrespective of the fact that models are forced by observed sea surface temperature. Therefore, there can be model biases in regional scale.


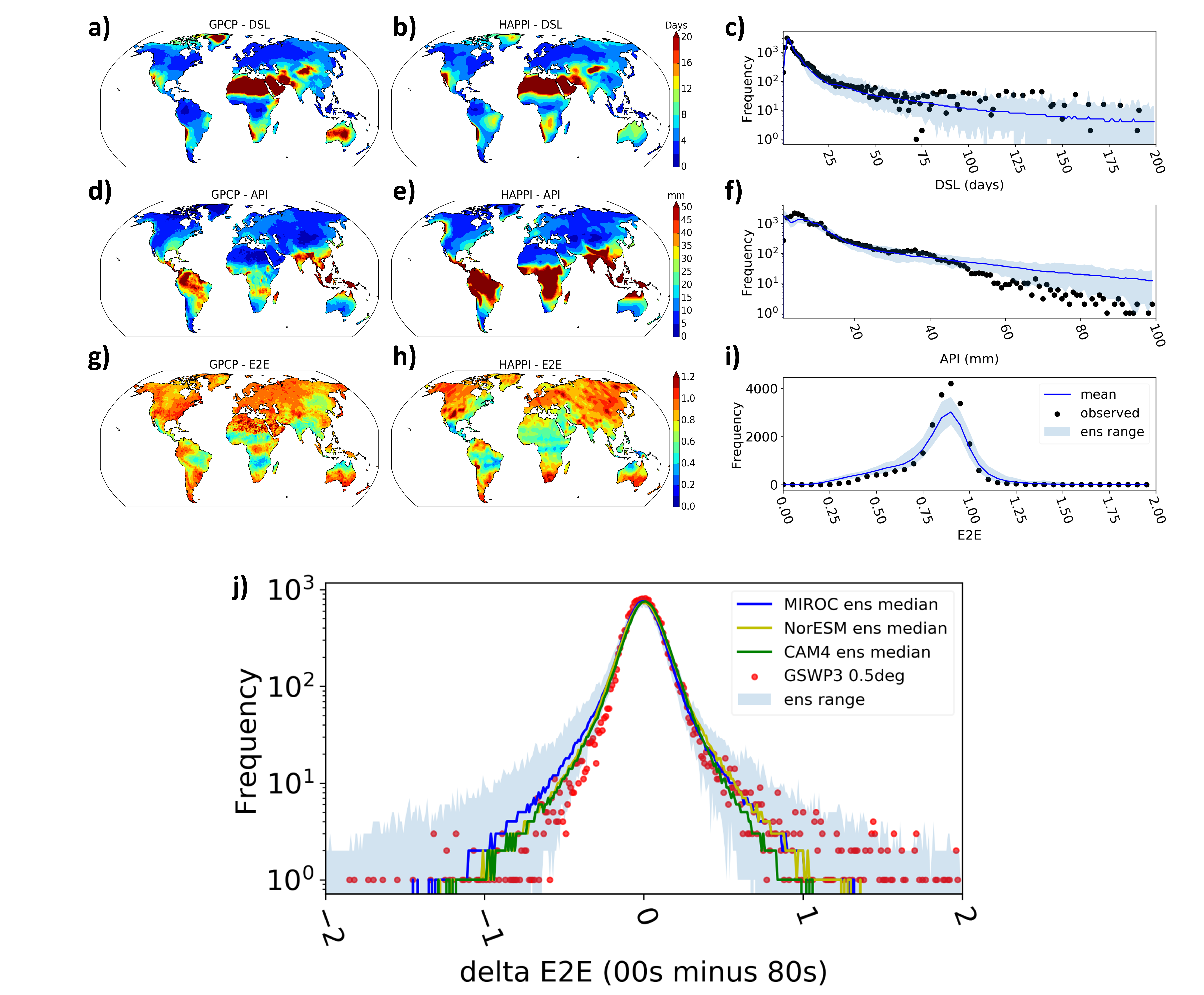


**Figure S2.** Spatial maps of average dry spell length (DSL), aggregated precipitation intensity (API), and event to event variability index (E2E) calculated for 2006-2015 for GPCP observed data^1^ (a,d,g) and HAPPI^2^ ALL simulation multimodel ensemble (b,e,h). Right panels (c,f,i) shows the comparison between GPCP and HAPPI ALL simulation multimodel ensemble of the occurrence of DSL, API and E2E values.Bottom panel (j) shows the comparison of E2E change between the periods periods 2001-2010 and 1979-1988, using GSWP3 observations^9^ and HAPPI simulations.


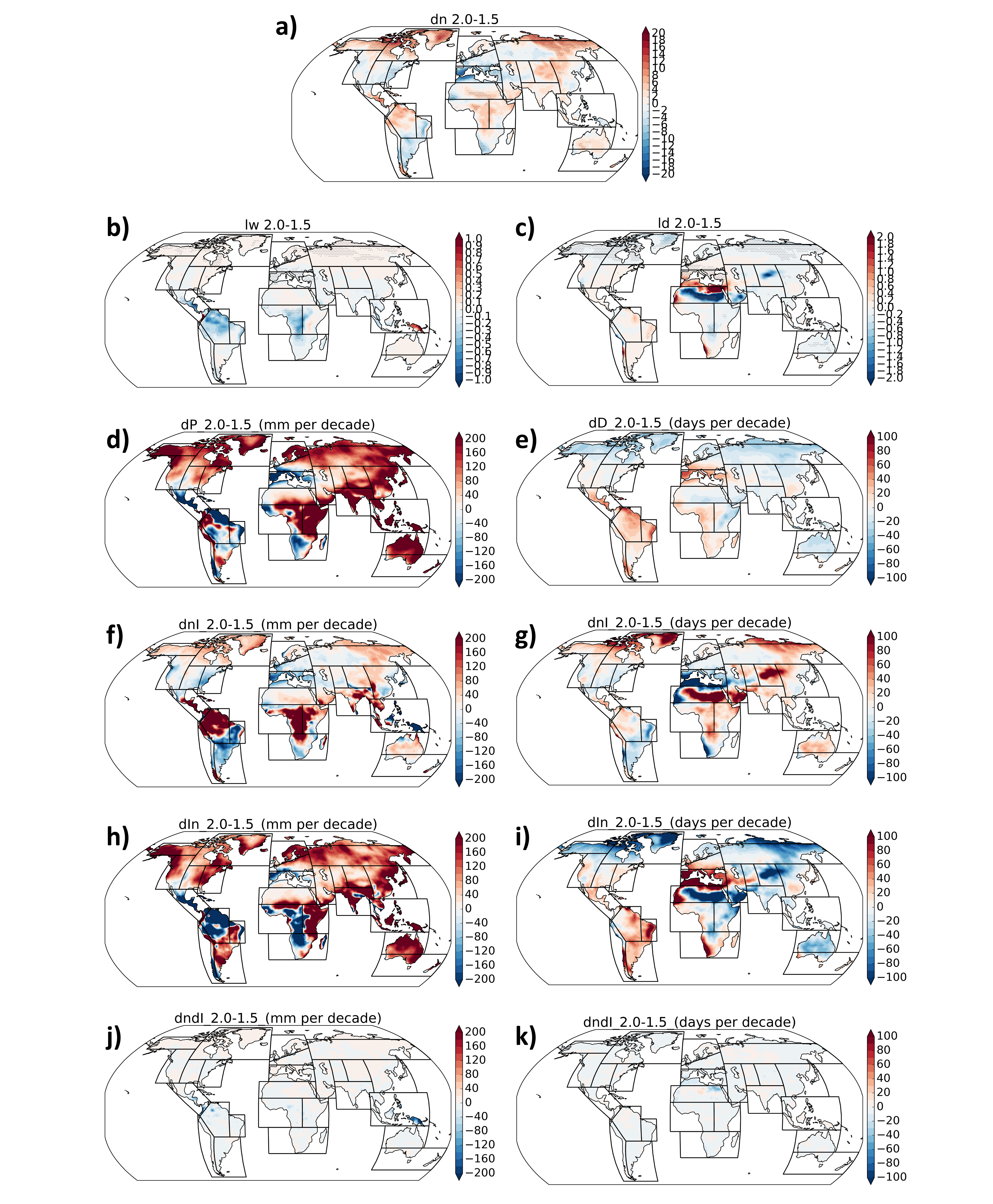


**Figure S3.** Global spatial map of 2 ^ο^C minus 1.5 ^ο^C HAPPI^2^ multimodel ensemble mean of number of **a)** number of events (dn), **b)** wet spell length (lw), **c)** dry spell length (ld), **d)** total precipitation during wet spells (dP), **e)** total dry days during dry spells (dD). **f**, **h**, **j** (**g, i, k**) are the frequency, intensity and covariance terms during wet spells (dry spells). DSL and API correspond to the intensity terms are shown in figure S6 (a,b). Black boxes represent the IPCC AR5 reference regions (<http://www.ipcc-data.org/guidelines/pages/ar5_regions.html>).


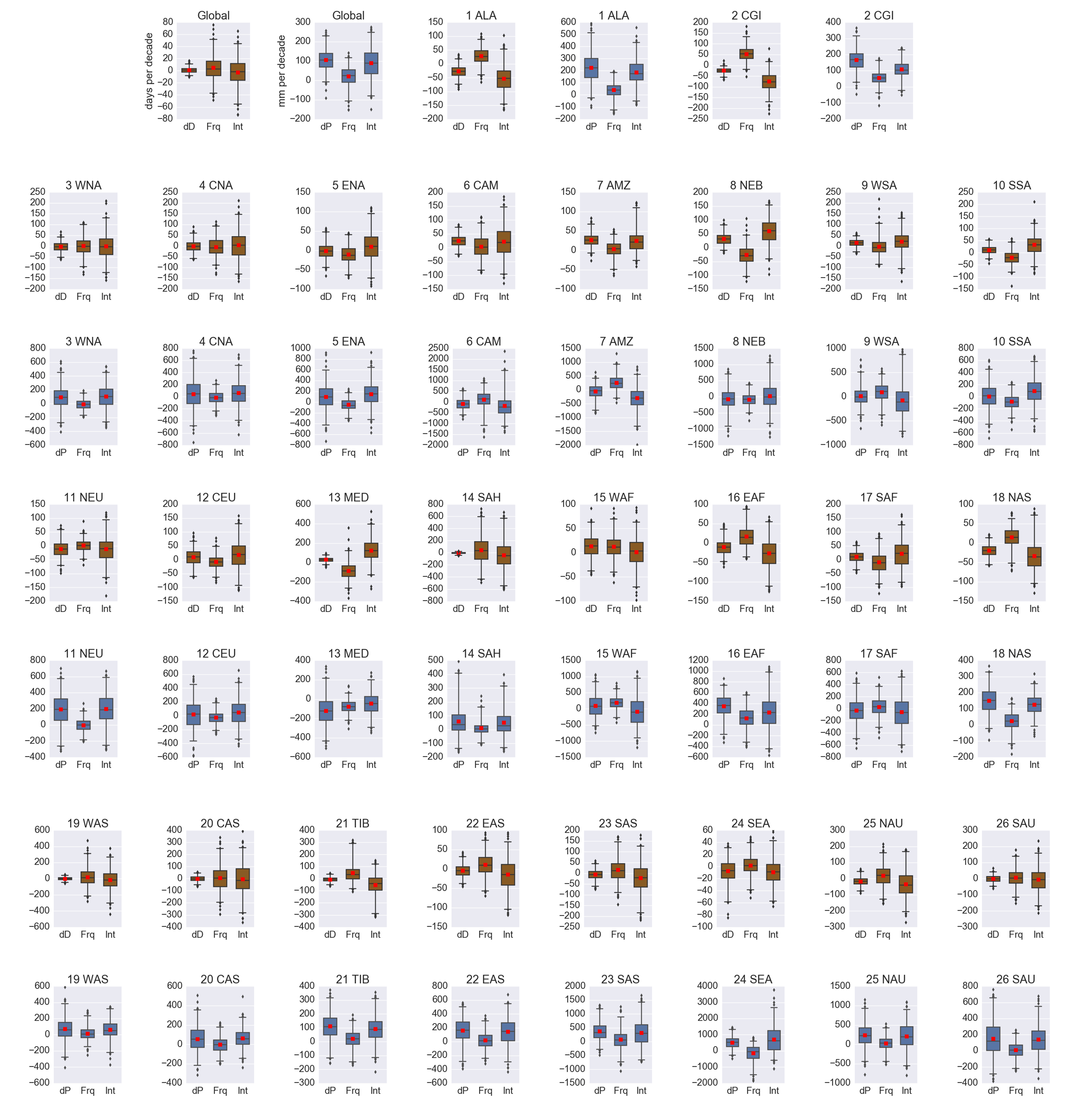


**Figure S4.** Box-whiskers plots show the area averaged (land only) HAPPI^2^ 2 ^ο^C minus 1.5 ^ο^C difference between 2 ^ο^C and 1.5 ^ο^C climates of total precipitation (dP) and total dry days (dD), frequency term (Frq) and intensity term (Int) during wet spells (blue) dry spells (brown) for global and 26 SREX regions. Variables for wet spells (dry spells) are in unit mm (days) per decade. Box-whiskers plots indicate the 10th, 25th, 50th, 75th and 90th percentiles and the rest as outliers. Red square in plots shows the mean value of the multimodel ensemble distribution.


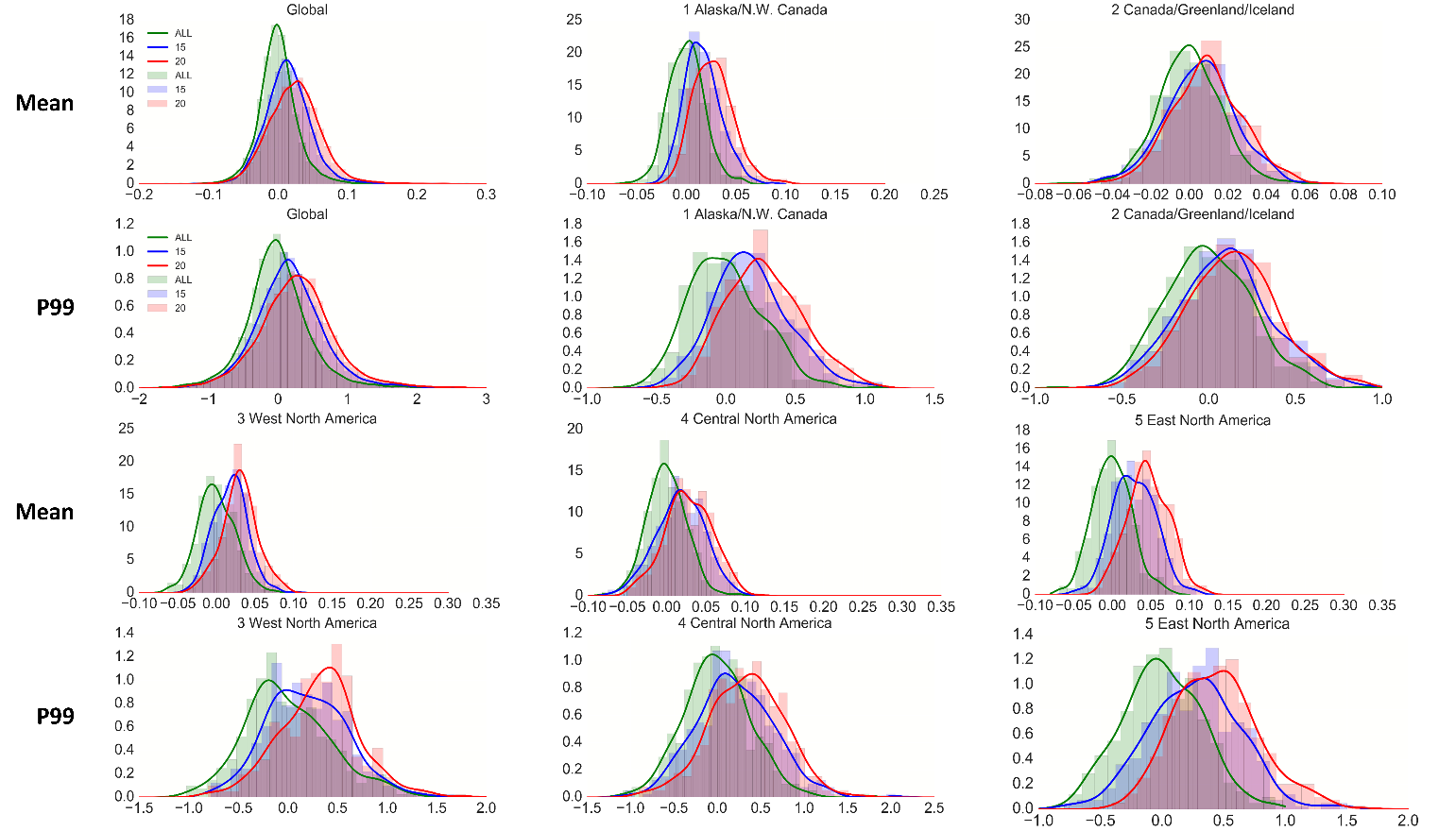


**Figure S5.** Histogram and kernel density estimation (KDE; thick line) of the area averaged (land only) E2E mean and P99 distribution of each region for HAPPI^2^ historical (ALL) period and 1.5 °C (15) and 2 °C (20) future scenarios for global and 26 SREX regions. ALL, 1.5 and 2 °C scenarios are shown by green, blue and red colors respectively. Bin width of the P99 (mean) histograms is set to 0.1 (0.01).


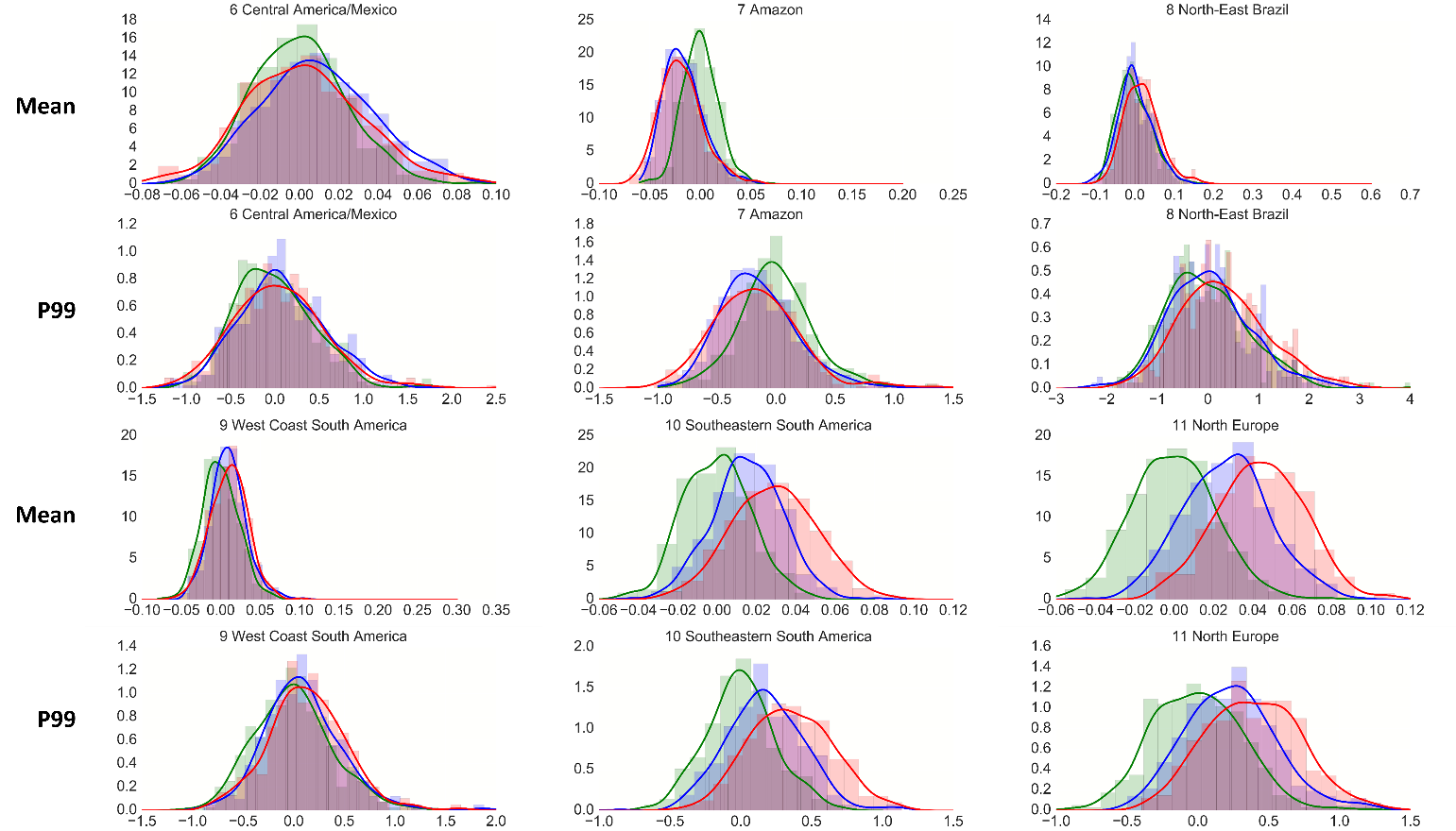


**Figure S5.** continued.


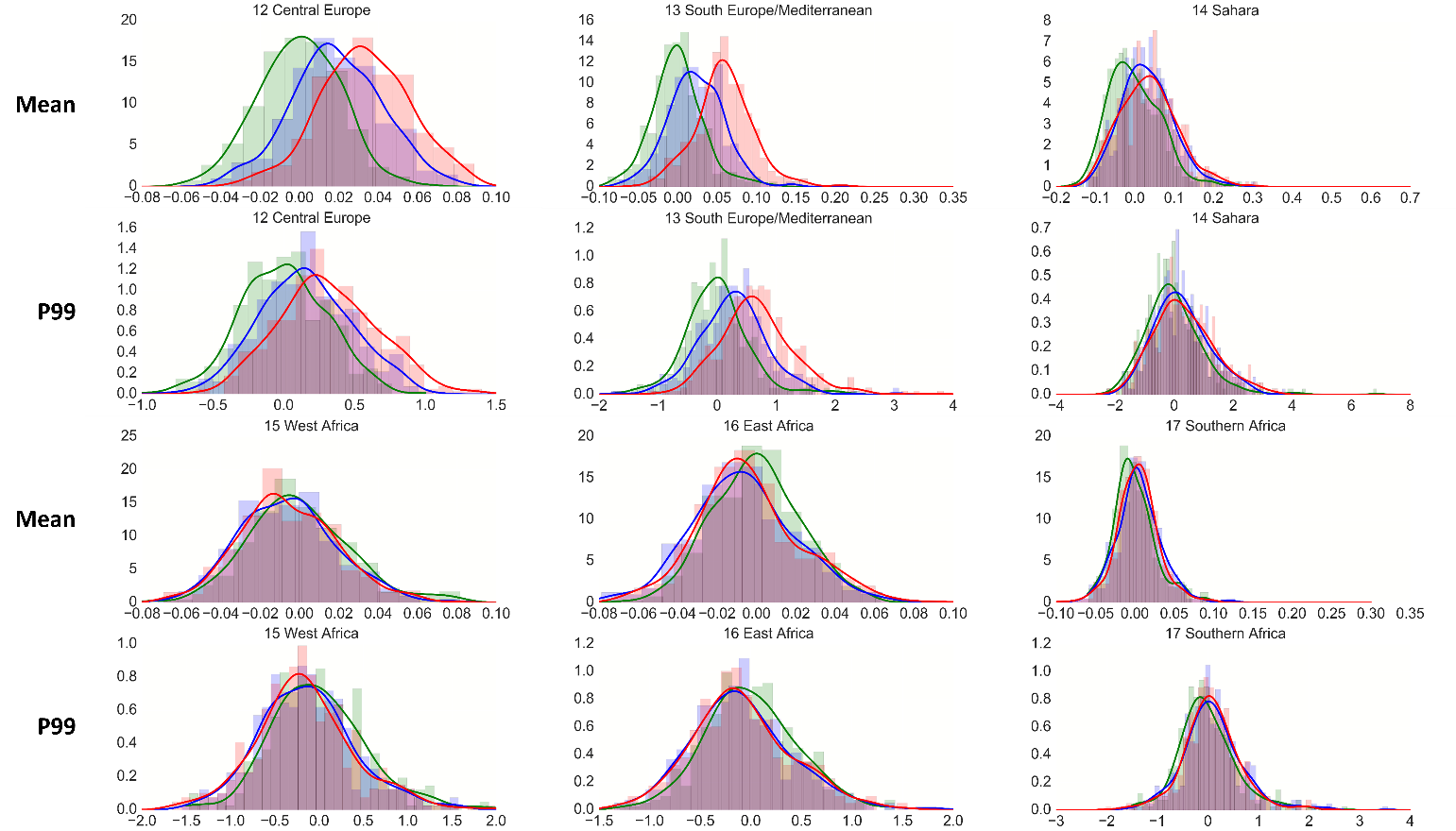


**Figure S5.** continued.


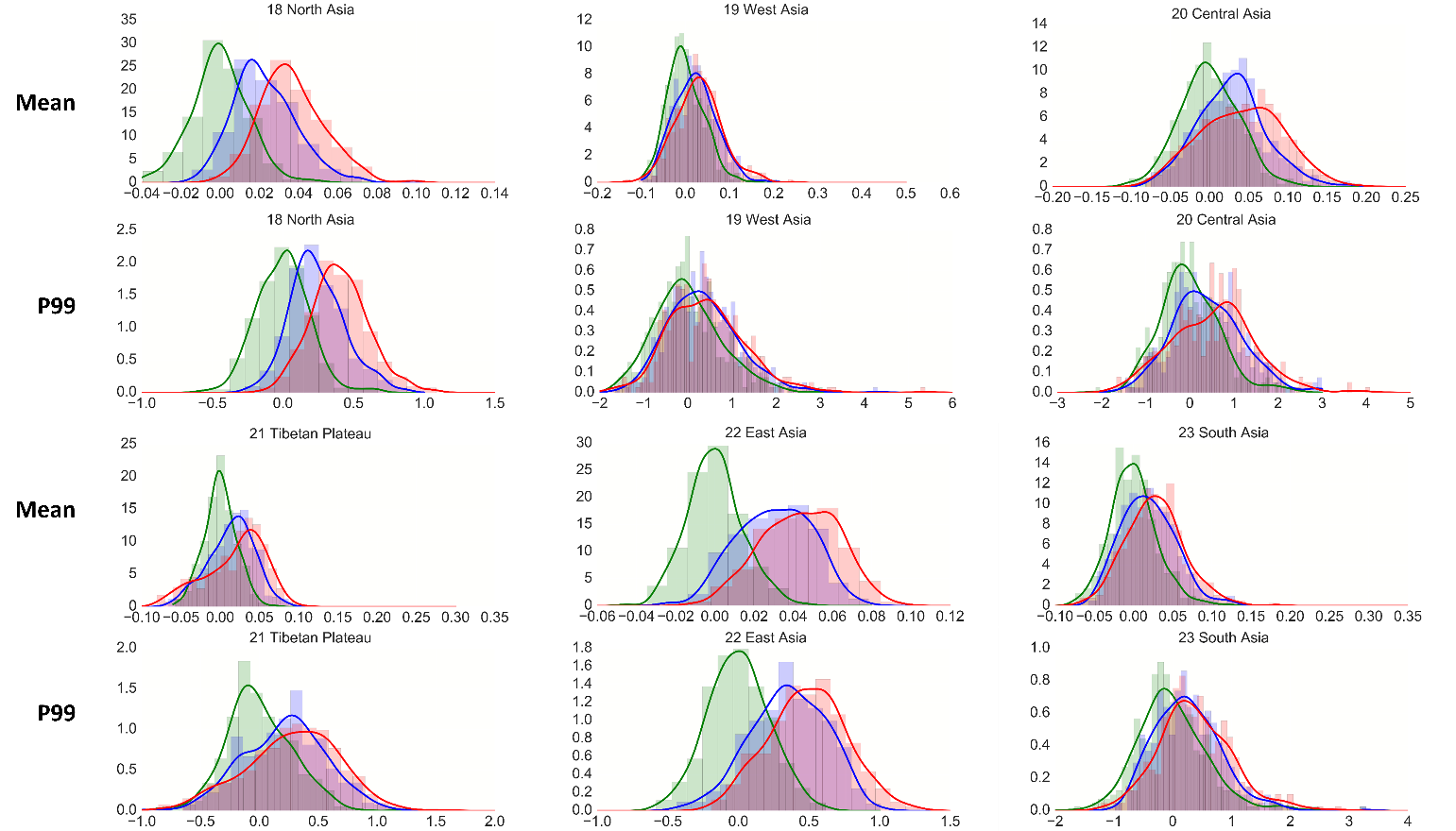

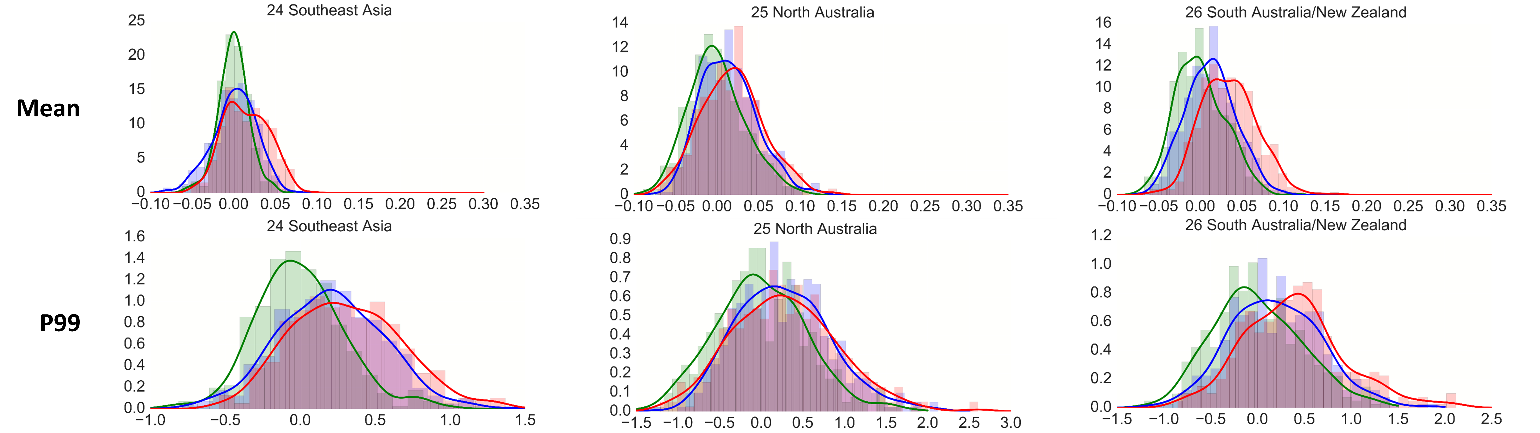


**Figure S5.** continued.

**Figure S6.** Global spatial map and zonal mean of the HAPPI^2^ 2°C minus 1.5°C DSL mean (a), DSL P99 (b), API mean (c) and API P99 (d) multi-model ensemble mean of the HAPPI data^5^ (95% significant level is stippled). Black boxes represent the IPCC AR5 reference regions (<http://www.ipcc-data.org/guidelines/pages/ar5_regions.html>).

DSL and API mean results correspond to the intensity terms (dIn) of dry and wet spells in figure S3, respectively, but with normalized values used to derive E2E.


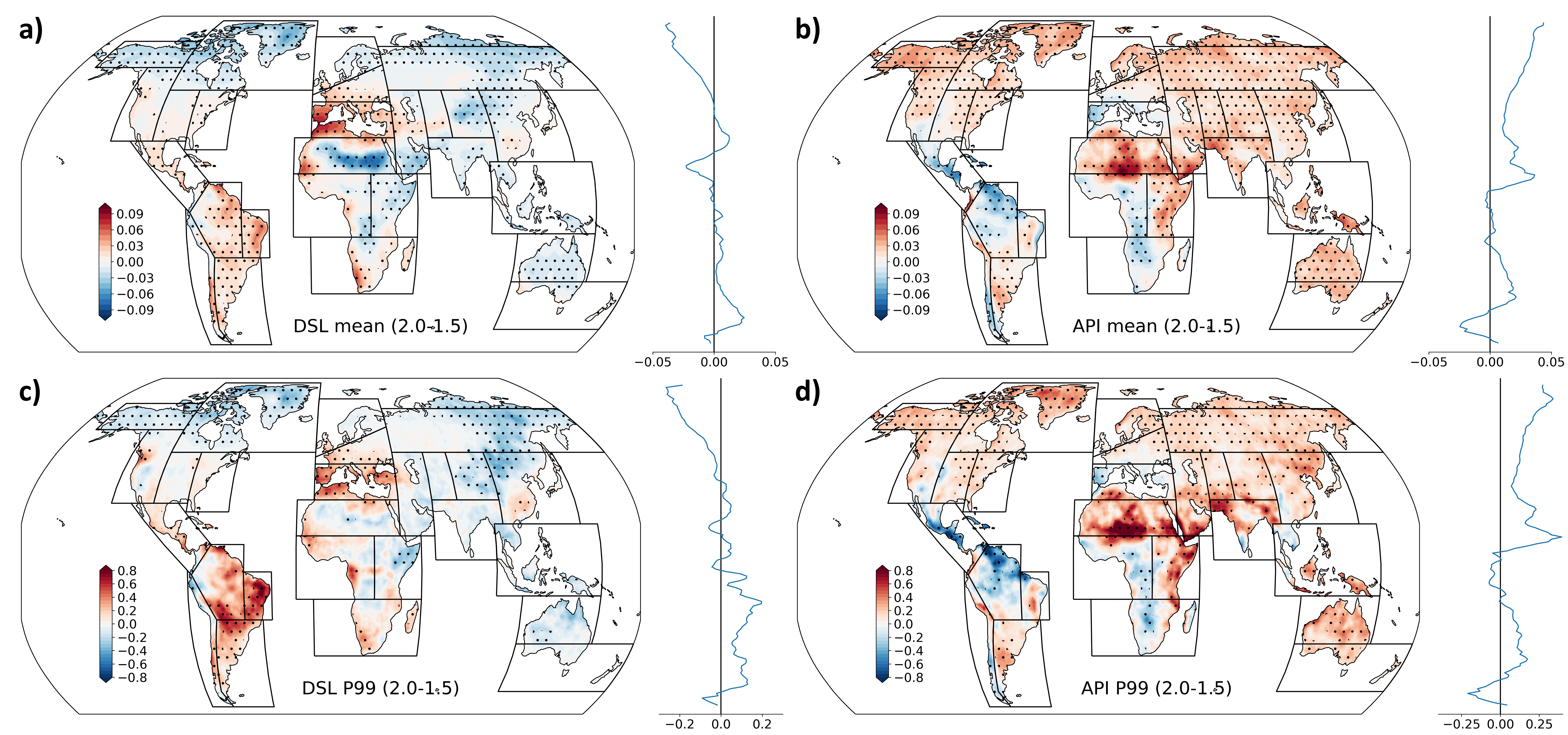


|  | **E2E mean anomaly** | | | | | | | | | | | |
| --- | --- | --- | --- | --- | --- | --- | --- | --- | --- | --- | --- | --- |
|  | **ALL** | | | | **15** | | | | **20** | | | |
|  | **mean** | **std** | **kurt** | **skew** | **mean** | **std** | **kurt** | **skew** | **mean** | **std** | **kurt** | **skew** |
| **Global** | 0.0000 | 0.0285 | 4.1915 | 0.6147 | 0.0139 | 0.0331 | 2.8930 | 0.5736 | 0.0236 | 0.0380 | 1.9531 | 0.4321 |
| **1 ALA** | 0.0000 | 0.0178 | 0.5093 | 0.1559 | 0.0163 | 0.0181 | 0.3166 | 0.4758 | 0.0271 | 0.0197 | 0.5055 | 0.5228 |
| **2 CGI** | 0.0000 | 0.0161 | 0.6961 | 0.0069 | 0.0058 | 0.0180 | 0.2287 | -0.0441 | 0.0089 | 0.0183 | 0.1372 | 0.0327 |
| **3 WNA** | 0.0000 | 0.0237 | 0.1035 | 0.1864 | 0.0183 | 0.0218 | 0.2548 | 0.1715 | 0.0303 | 0.0233 | 0.2539 | -0.0122 |
| **4 CNA** | 0.0000 | 0.0251 | 0.6659 | 0.1842 | 0.0173 | 0.0306 | -0.0985 | -0.1541 | 0.0283 | 0.0302 | -0.2531 | -0.0622 |
| **5 ENA** | 0.0000 | 0.0251 | 0.1589 | 0.1589 | 0.0284 | 0.0275 | -0.0381 | 0.0870 | 0.0465 | 0.0267 | -0.2716 | 0.0595 |
| **6 CAM** | 0.0000 | 0.0235 | 0.1443 | 0.2843 | 0.0091 | 0.0283 | -0.2132 | 0.1955 | 0.0025 | 0.0297 | 0.0595 | 0.2368 |
| **7 AMZ** | 0.0000 | 0.0174 | 0.6496 | 0.1710 | -0.0163 | 0.0195 | 0.6746 | 0.7373 | -0.0183 | 0.0216 | 0.5563 | 0.5685 |
| **8 NEB** | 0.0000 | 0.0428 | 0.1898 | 0.6010 | 0.0015 | 0.0429 | 0.1656 | 0.2683 | 0.0181 | 0.0450 | 0.6707 | 0.5633 |
| **9 WSA** | 0.0000 | 0.0227 | 0.0911 | 0.1937 | 0.0098 | 0.0223 | 1.0995 | 0.5282 | 0.0109 | 0.0236 | 0.3402 | 0.1032 |
| **10 SSA** | 0.0000 | 0.0169 | -0.0337 | 0.0893 | 0.0154 | 0.0178 | 0.3205 | 0.0001 | 0.0290 | 0.0212 | -0.1998 | 0.0311 |
| **11 NEU** | 0.0000 | 0.0210 | 0.1977 | 0.1970 | 0.0265 | 0.0218 | -0.0534 | 0.0480 | 0.0439 | 0.0221 | -0.1485 | 0.0139 |
| **12 CEU** | 0.0000 | 0.0207 | -0.0345 | -0.0365 | 0.0188 | 0.0230 | -0.1423 | 0.0072 | 0.0332 | 0.0230 | -0.0873 | -0.0821 |
| **13 MED** | 0.0000 | 0.0330 | 1.1830 | 0.4897 | 0.0227 | 0.0347 | 0.5388 | 0.1424 | 0.0579 | 0.0376 | 1.2521 | 0.2711 |
| **14 SAH** | 0.0000 | 0.0658 | 0.7793 | 0.6775 | 0.0316 | 0.0667 | 0.7222 | 0.5259 | 0.0342 | 0.0733 | 0.3924 | 0.4837 |
| **15 WAF** | 0.0000 | 0.0260 | 0.5557 | 0.4290 | -0.0058 | 0.0247 | 0.1542 | 0.3186 | -0.0056 | 0.0247 | 0.2706 | 0.2223 |
| **16 EAF** | 0.0000 | 0.0224 | 0.1278 | 0.1638 | -0.0068 | 0.0259 | 0.0967 | 0.1490 | -0.0045 | 0.0265 | 0.4158 | 0.3631 |
| **17 SAF** | 0.0000 | 0.0265 | 1.0347 | 0.7326 | 0.0055 | 0.0284 | 1.4841 | 0.5125 | 0.0041 | 0.0265 | 1.4560 | 0.2480 |
| **18 NAS** | 0.0000 | 0.0144 | 0.5760 | 0.1408 | 0.0225 | 0.0157 | 0.1896 | 0.4883 | 0.0369 | 0.0163 | 0.4359 | 0.4671 |
| **19 WAS** | 0.0000 | 0.0410 | 0.2197 | 0.4395 | 0.0219 | 0.0480 | 0.2579 | 0.3532 | 0.0298 | 0.0530 | 0.7150 | 0.3084 |
| **20 CAS** | 0.0000 | 0.0372 | 0.0497 | 0.0931 | 0.0283 | 0.0431 | 0.3650 | 0.2990 | 0.0417 | 0.0516 | -0.4892 | 0.0029 |
| **21 TIB** | 0.0000 | 0.0204 | 0.1276 | 0.0687 | 0.0142 | 0.0291 | -0.1980 | -0.3040 | 0.0193 | 0.0377 | -0.3544 | -0.6143 |
| **22 EAS** | 0.0000 | 0.0140 | 0.2415 | 0.2416 | 0.0303 | 0.0189 | -0.5049 | -0.1465 | 0.0437 | 0.0197 | -0.4715 | -0.1313 |
| **23 SAS** | 0.0000 | 0.0297 | 0.9228 | 0.4855 | 0.0187 | 0.0341 | 0.0379 | 0.3984 | 0.0277 | 0.0372 | 0.5581 | 0.3579 |
| **24 SEA** | 0.0000 | 0.0184 | 1.0818 | -0.0069 | 0.0012 | 0.0260 | 0.1171 | -0.3618 | 0.0152 | 0.0267 | -0.5947 | 0.0783 |
| **25 NAU** | 0.0000 | 0.0338 | 0.0879 | 0.3849 | 0.0138 | 0.0334 | 0.0845 | 0.4422 | 0.0186 | 0.0374 | 0.0549 | 0.2214 |
| **26 SAU** | 0.0000 | 0.0299 | -0.0116 | 0.3644 | 0.0122 | 0.0301 | -0.2318 | 0.1271 | 0.0347 | 0.0332 | 0.2771 | 0.3094 |

**Table S1.** Mean, standard deviation (std), kurtosis (kurt) and skewness (skew) of the distribution of the anomaly of E2E shown in Figure S5.

**Table S2.** Same as Table S1 but for P99 of E2E.

|  | **E2E P99 anomaly** | | | | | | | | | | | |
| --- | --- | --- | --- | --- | --- | --- | --- | --- | --- | --- | --- | --- |
|  | **ALL** | | | | **15** | | | | **20** | | | |
|  | **mean** | **std** | **kurt** | **skew** | **mean** | **std** | **kurt** | **skew** | **mean** | **std** | **kurt** | **skew** |
| **Global** | 0.0000 | 0.4903 | 8.3068 | 1.0409 | 0.1701 | 0.5258 | 3.0561 | 0.6121 | 0.2764 | 0.5800 | 3.1091 | 0.5845 |
| **1 ALA** | 0.0000 | 0.2666 | 0.3371 | 0.4456 | 0.1806 | 0.2749 | 0.3124 | 0.4171 | 0.3232 | 0.3162 | 0.6203 | -0.2314 |
| **2 CGI** | 0.0000 | 0.2472 | 0.1799 | 0.1321 | 0.0976 | 0.2713 | 0.1337 | 0.2023 | 0.1659 | 0.3131 | 0.5742 | 0.1749 |
| **3 WNA** | 0.0000 | 0.4242 | 0.0407 | 0.4257 | 0.1886 | 0.4006 | 0.1455 | 0.2939 | 0.3079 | 0.3948 | 0.4109 | -0.1299 |
| **4 CNA** | 0.0000 | 0.3822 | 0.8058 | 0.2669 | 0.1990 | 0.4372 | 0.3608 | 0.2146 | 0.3934 | 0.4021 | 0.1093 | -0.1230 |
| **5 ENA** | 0.0000 | 0.3187 | -0.1767 | 0.0918 | 0.2642 | 0.3679 | 0.2567 | 0.0815 | 0.4048 | 0.3723 | 0.5192 | 0.0409 |
| **6 CAM** | 0.0000 | 0.4492 | 0.8618 | 0.6229 | 0.0859 | 0.4845 | 0.0989 | 0.3073 | 0.1369 | 0.5298 | 0.8898 | 0.3402 |
| **7 AMZ** | 0.0000 | 0.3219 | 1.7002 | 0.5249 | -0.1500 | 0.3285 | 1.5692 | 0.7785 | -0.0747 | 0.4620 | 0.0103 | 0.5347 |
| **8 NEB** | 0.0000 | 0.8184 | 1.4628 | 0.7728 | 0.0408 | 0.8102 | 0.3932 | 0.3717 | 0.3058 | 0.7948 | 0.4999 | 0.3652 |
| **9 WSA** | 0.0000 | 0.3830 | 0.3412 | 0.3748 | 0.0890 | 0.3799 | 2.0753 | 0.7298 | 0.1680 | 0.3890 | 0.6310 | 0.1782 |
| **10 SSA** | 0.0000 | 0.2418 | 0.0899 | 0.1152 | 0.1725 | 0.2736 | 0.6961 | 0.2028 | 0.3751 | 0.3254 | 0.5970 | -0.3224 |
| **11 NEU** | 0.0000 | 0.3154 | 0.3503 | 0.2824 | 0.2368 | 0.3177 | 0.2758 | 0.3973 | 0.3975 | 0.3674 | 0.1055 | -0.1890 |
| **12 CEU** | 0.0000 | 0.3026 | -0.0294 | -0.0009 | 0.1544 | 0.3228 | -0.2383 | 0.0706 | 0.3099 | 0.3797 | -0.0629 | -0.0259 |
| **13 MED** | 0.0000 | 0.5546 | 2.4935 | 0.6563 | 0.2737 | 0.5950 | 2.3099 | 0.3850 | 0.5840 | 0.6546 | 2.5548 | 0.7985 |
| **14 SAH** | 0.0000 | 1.0043 | 6.4454 | 1.5122 | 0.2524 | 0.9353 | 0.1086 | 0.4799 | 0.3525 | 0.8978 | 0.9709 | 0.4630 |
| **15 WAF** | 0.0000 | 0.5395 | 0.7577 | 0.4712 | -0.1349 | 0.5387 | 0.4157 | 0.3985 | -0.1221 | 0.5875 | 0.3416 | 0.2059 |
| **16 EAF** | 0.0000 | 0.4508 | 0.1587 | 0.1915 | -0.0894 | 0.5001 | 0.6556 | 0.4854 | -0.0075 | 0.4957 | -0.1845 | 0.0755 |
| **17 SAF** | 0.0000 | 0.5867 | 2.1348 | 0.8330 | 0.0999 | 0.6253 | 3.4452 | 0.9330 | 0.1228 | 0.6111 | 1.5248 | 0.1952 |
| **18 NAS** | 0.0000 | 0.1852 | 0.7974 | 0.2640 | 0.2440 | 0.1939 | 0.3144 | 0.4449 | 0.4074 | 0.2829 | 2.4617 | -0.8175 |
| **19 WAS** | 0.0000 | 0.7567 | 0.4360 | 0.5220 | 0.3401 | 0.8035 | 1.2408 | 0.6616 | 0.4689 | 0.7245 | 1.1463 | 0.7114 |
| **20 CAS** | 0.0000 | 0.6749 | 0.9355 | 0.5294 | 0.3903 | 0.7921 | 0.3573 | 0.3842 | 0.6509 | 0.7651 | 1.6383 | 0.5996 |
| **21 TIB** | 0.0000 | 0.2803 | 0.7996 | 0.3103 | 0.2137 | 0.3483 | 0.0284 | -0.0806 | 0.3867 | 0.3469 | 0.6829 | -0.3480 |
| **22 EAS** | 0.0000 | 0.2167 | -0.1294 | 0.0575 | 0.3536 | 0.2668 | -0.3272 | -0.2356 | 0.5005 | 0.3234 | 1.7262 | -0.7827 |
| **23 SAS** | 0.0000 | 0.6026 | 2.8971 | 0.9569 | 0.2533 | 0.5590 | 1.7821 | 0.7581 | 0.4351 | 0.6181 | 1.1306 | 0.5245 |
| **24 SEA** | 0.0000 | 0.2951 | 0.7226 | 0.3589 | 0.2046 | 0.3525 | 0.1256 | 0.1234 | 0.3599 | 0.3824 | 0.0326 | -0.1469 |
| **25 NAU** | 0.0000 | 0.5495 | 0.2178 | 0.2734 | 0.2684 | 0.5656 | 0.1646 | 0.4070 | 0.3570 | 0.5850 | 0.6336 | 0.3462 |
| **26 SAU** | 0.0000 | 0.4745 | -0.1430 | 0.3282 | 0.1927 | 0.4862 | 0.1246 | 0.2170 | 0.3900 | 0.5345 | 0.4929 | 0.4798 |

**References**

1. Huffman, G. J. *et al.* Global precipitation at one-degree daily resolution from multisatellite observations. *J. Hydrometeorol.* **2**, 36-50 (2001).
2. Mitchell, D. *et al.* Half a degree additional warming, prognosis and projected impacts (HAPPI): background and experimental design. *Geosci. Model Dev.* **10**, 571 (2017).
3. Liu, J. *et al.* What drives the global summer monsoon over the past millennium? *Clim. Dyn.* **39**, 1063-1072 (2012).
4. Wang, P. X. *et al.* The global monsoon across timescales: coherent variability of regional monsoons. *Clim. Past*, **10**, 2007 (2014).
5. Chen, D. & Chen, H. W. Using the Köppen classification to quantify climate variation and change: an example for 1901–2010. *Environ. Dev.* **6**, 69-79 (2013).
6. Chevuturi, A., Klingaman, N. P., Turner, A. G. & Hannah, S. Projected Changes in the Asian-Australian Monsoon Region in 1.5°C and 2.0°C Global-Warming Scenarios. *Earth's Future* **6**, 339-358 (2018).
7. Coats, S., Smerdon, J. E., Cook, B. I., Seager, R., Cook, E. R. & Anchukaitis, K. J. Internal ocean-atmosphere variability drives megadroughts in Western North America. *Geophys. Res. Lett.* **43**, 9886-9894 (2016).
8. Lehner, F. *et al.* Projected drought risk in 1.5°C and 2°C warmer climates, *Geophys. Res. Lett.* **44**, 7419–7428 (2017).
9. Kim, H. Global Soil Wetness Project Phase 3 Atmospheric Boundary Conditions (Experiment 1) [Data set]. Data Integration and Analysis System (DIAS). <https://doi.org/10.20783/DIAS.501> (2017).
